# Population pharmacokinetic modeling in radiopharmaceutical therapy: a review

**DOI:** 10.3389/fnume.2025.1695332

**Published:** 2025-10-09

**Authors:** Deni Hardiansyah, Bisma Barron Patrianesha, Gerhard Glatting

**Affiliations:** ^1^ Faculty of Mathematics and Natural Sciences, Medical Physics and Biophysics Division, Physics Department, Universitas Indonesia, Depok, Indonesia; ^2^ Research Center for Safety, Metrology, and Nuclear Quality Technology, National Research and Innovation Agency (BRIN), KST B.J. Habibie, Tangerang Selatan, Indonesia; ^3^ Medical Radiation Physics, Department of Nuclear Medicine, Ulm University, Ulm, Germany

**Keywords:** POPPK, RPT, NLMEM, STP, PKPD

## Abstract

Population pharmacokinetic (PopPK) has emerged as a robust framework for characterizing inter-individual variability in the absorbed dose estimates in radiopharmaceutical therapy (RPT). By enabling the analysis of biokinetic data from heterogeneous patient populations, PopPK allows individualized absorbed dose estimates while simultaneously leveraging population-level information. This review presents and evaluates the current applications of PopPK, such as nonlinear mixed-effects modeling (NLMEM) and Bayesian fitting methods in RPT, emphasizing its advantages over traditional individual-based modeling approaches. We summarize key studies that have implemented PopPK for modeling radiopharmaceutical biokinetics, with a focus on time-integrated activity (TIA) estimation, including single-time-point (STP) dosimetry, uncertainty analysis, as well as pharmacodynamic (PD) analysis. The flexibility of PopPK in handling sparse and irregularly sampled data makes it particularly relevant for clinical scenarios where comprehensive imaging schedules are impractical. However, despite its potential, the widespread adoption of PopPK in RPT remains limited due to challenges such as computational complexity and the need for specialized expertise. This review discusses critical aspects of PopPK implementation while emphasizing the importance of interdisciplinary collaboration in translating PopPK methodologies into clinical practice. Future directions include integrating PopPK into adaptive dosimetry frameworks and applying it in STP dosimetry and PD modeling to optimize treatment personalization. By providing a comprehensive overview of PopPK applications in RPT, this review aims to facilitate the integration of advanced modeling techniques into routine clinical workflows, ultimately supporting the development of accurate and precise RPTs.

## 1 Introduction

Radiopharmaceutical therapy (RPT) is commonly administered using fixed activity protocols or simple adjustments based on body surface area (1–3). However, evidence shows that absorbed doses can differ markedly between patients receiving the same administered activity, raising the risk of suboptimal tumor control or avoidable toxicity (3–12). Recognizing this, the European Council Directive 2013/59/EURATOM mandates patient-specific treatment planning and verification for all radiotherapeutic procedures, underscoring the necessity of individualized dosimetry approaches that capture inter-individual variability in radiopharmaceutical distribution and clearance (13).

A central challenge in individualized dosimetry is the reliable estimation of time-integrated activity (TIA) from time–activity curves (TACs), particularly when imaging measurement data are sparse, a frequent condition in nuclear medicine practice. Incorporating prior knowledge into the TAC fitting process has been shown to improve the accuracy and precision of TIA and, consequently, absorbed dose estimates (14–17). Population pharmacokinetic (PopPK) modeling offers a powerful framework for achieving this. Unlike conventional individual fitting approaches, PopPK leverages data sharing across patients, fitting model parameters simultaneously at the population level while still yielding individualized estimates. This paradigm enhances the ratio of data to estimated parameters, thereby improving the accuracy of TIA estimates. Importantly, PopPK, specifically nonlinear mixed-effects modeling (NLMEM), is recognized by both the FDA and EMA as standard methodologies in drug development, particularly valuable for handling sparse data and heterogeneous measurement protocols (18, 19).

Several pioneering studies have highlighted the value of PopPK modeling in RPT. Hardiansyah et al. provided the first demonstrations that integration of population priors within PopPK framework significantly strengthens individual dosimetry in RPT (20). Merril et al. optimized sampling schedules for ^131^I therapy in Graves' disease (21), while Puszkiel et al. employed a three-compartment PopPK model to quantify the effects of amino acid co-infusion on [^177^Lu]Lu-DOTATATE pharmacokinetics and toxicity in neuroendocrine tumor patients (22). Devasia et al. (17) applied a bi-exponential function within NLMEM for single-time-point (STP) dosimetry of [¹⁷⁷Lu]Lu-DOTATATE. They demonstrated reduced bias compared to the commonly used STP approach proposed by Hänscheid (23) and Madsen (24). Hardiansyah et al. introduced population-based model selection (PBMS) with PopPK modeling to improve the accuracy of absorbed dose estimates (25). More recently, Hardiansyah et al. introduced a population-based model selection framework within the NLMEM paradigm to optimize sum-of-exponential functions for ^131^I therapy in benign thyroid disease, demonstrating superior accuracy compared with standard individual fitting approaches recommended by the European Association of Nuclear Medicine (15). Collectively, these studies demonstrate the ability of NLMEM to combine population-level priors with patient-specific data, thereby enabling accurate and robust absorbed dose estimation. The methodological rigor and flexibility of NLMEM make it particularly well suited for routine clinical use, where simplified protocols and sparse data are the norm. Examples of NLMEM applications in RPT, along with software suitable for NLMEM analyses, are provided in the supplemental file (Supplementary Tables S1, S2).

## 2 Clinical relevance of PopPK in RPT

One of the major challenges in RPT dosimetry is the absence of a standardized approach for selecting fit functions to calculate TIAs, a critical determinant of absorbed dose. In current practice, fit function selection is often guided by subjective “rules of thumb”, primarily based on the number of available biokinetic data points. As highlighted in an EANM recommendation (26), at least three data points are required to fit a mono-exponential function with two estimated parameters in individual fitting methods. This aligns with the general principle outlined by Gear et al. (27), which emphasizes that the number of data points should exceed the number of parameters to avoid overfitting and ensure reliable uncertainty estimation.

Typically, radiopharmaceutical kinetics exhibit multiphasic behavior, including an uptake phase and multiple clearance phases (28, 29). Due to limited data availability and the reliance on subjective modeler judgment, simplified mono-exponential or, at best, bi-exponential functions are often employed (30–32). Such subjective choices may introduce substantial variability in absorbed dose estimates and bias dose–effect relationships, ultimately undermining the reliability of clinical dosimetry.

Population pharmacokinetic (PopPK) modeling provides a systematic and objective alternative for analyzing radiopharmaceutical kinetics. Rather than relying on subjective judgment, fit functions can be identified through PBMS, which applies robust statistical criteria to determine the optimal model. This approach provides several advantages:

1. objectivity, relying on statistical measures such as goodness-of-fit tests and Akaike weights (33, 34);
2. systematic evaluation, testing a range of models from simple single-phase to more complex multi-phase functions (35); PBMS NLMEM can also be used to test various structures of compartmental and physiologically-based pharmacokinetic models.
3. accuracy, as PBMS NLMEM has been shown to outperform rule-of-thumb or individual-based model selection (IBMS) approaches (36), including parameter-sharing PBMS strategies (14, 28); and
4. reproducibility, since model choice is determined by the data and not by subjective judgment of the modeler, leading to more consistent TIA and absorbed dose estimates.

A key strength of PBMS within the NLMEM framework is its ability to pool biokinetic data from across patients. This increases the ratio of observations (N) to estimated parameters (K), enabling the construction of more complex yet stable models, while simultaneously reducing uncertainty in model selection. Importantly, this framework is particularly powerful under conditions of sparse sampling, a common condition in clinical nuclear medicine, where it could maintain good accuracy despite limited data (15, 25, 35, 37, 38). Given these advantages, PBMS–NLMEM holds strong potential not only for improving individualized dosimetry but also for serving as a robust reference framework system in RPT, ensuring standardization, reproducibility, and regulatory confidence.

## 3 Pharmacodynamic analysis

PopPK and pharmacodynamic (PD) modeling have emerged as powerful approaches to characterize drug behavior across heterogeneous patient populations (39–41). By quantifying inter-individual variability and identifying covariates that influence drug disposition and response, these models are increasingly recognized as essential tools for optimizing dosing strategies and improving clinical outcomes in RPT. Recent studies have demonstrated the value of population PK/PD modeling in characterizing the kinetics of [^177^Lu]Lu-labeled radiopharmaceuticals and explaining inter-patient variability in therapeutic outcomes (22, 42).

In one study, a PopPK model was applied to evaluate the impact of amino acid (AA) co-infusion on the pharmacokinetics of [^177^Lu]Lu-DOTATATE in patients with gastroenteropancreatic neuroendocrine tumors (22). Using a three-compartment model, investigators found that AA co-infusion significantly increased the elimination rate constant (k₁₀), with substantial inter-individual variability (104%). This variability translated into differences in systemic exposure, which were associated with hematological toxicity, particularly lymphopenia observed on Day 15. Notably, the population-based framework enabled identification of covariate effects and highlighted the need for personalized supportive care strategies in peptide receptor radionuclide therapy.

A complementary study extended PopPK analysis to [^177^Lu]Lu-PSMA-I&T in patients with metastatic castration-resistant prostate cancer (mCRPC). Here, a five-compartment model informed by quantitative SPECT/CT data was developed to describe radiopharmaceutical uptake in tumors and normal organs (42). The model was subsequently integrated into a PK/PD framework by linking tumor-level drug concentrations to longitudinal PSA dynamics. As in the DOTATATE study, pronounced inter-individual variability in tumor uptake was observed, with a progressive decline across treatment cycles. PSA response was captured using both direct and delayed drug-effect models, with tumor exposure emerging as a strong predictor of therapeutic efficacy. The incorporation of covariates such as renal function, tumor volume, and cycle number provided additional mechanistic insight into variability in treatment response.

Together, these studies underscore the clinical relevance of population PK/PD modeling in advancing precision medicine for RPT. Despite differences in radiopharmaceuticals, clinical indications, and endpoints, both investigations underscore the central role of individualized modeling in unraveling the complex interplay between drug exposure, biological response, and patient-specific factors. By enabling more accurate prediction of efficacy and toxicity, population PK/PD modeling supports the rational design of tailored dosing regimens, ultimately improving the therapeutic index of [^177^Lu]Lu-based radiopharmaceutical therapies.

## 4 Simplified dosimetry

**Figure 1:**
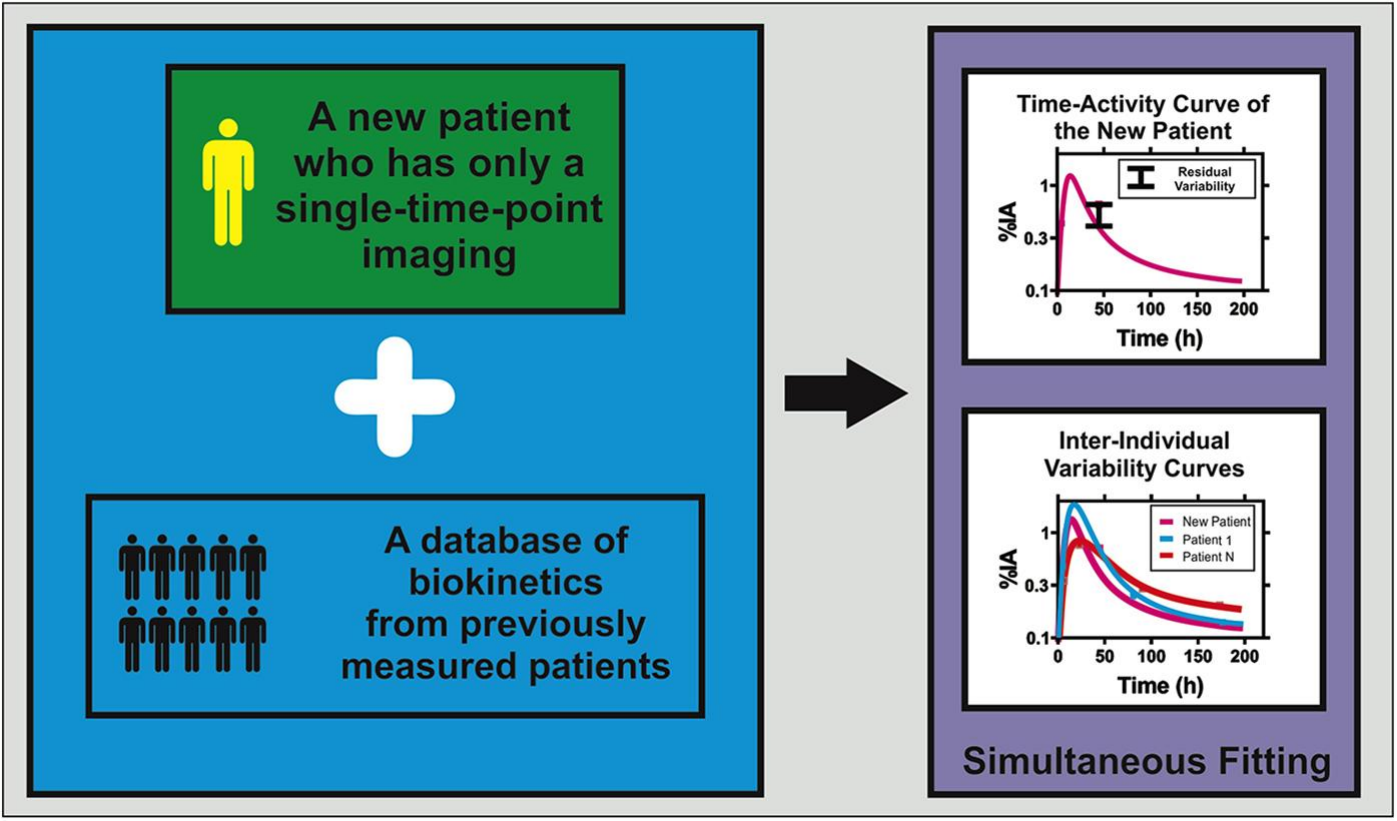
Conceptual framework of population pharmacokinetic modeling for single-time-point dosimetry in radiopharmaceutical therapy. The approach combines biokinetic data from a new patient with single-time-point imaging with a comprehensive biokinetic database from previous population studies. Through simultaneous fitting using nonlinear mixed-effects modeling, the method generates individualized time-integrated activity coefficients (TIACs) for the new patient while accounting for inter-individual variability observed in the population. The upper graph shows the predicted time-activity curve for the new patient, along with associated residual variability bounds. The lower graph demonstrates the inter-individual variability curves from the population database, illustrating how population pharmacokinetic modeling leverages shared information to enable accurate absorbed dose estimation using minimal individual patient data.

PopPK provides a robust framework for simplified dosimetry in RPT (15, 17, 25, 35, 38). By estimating mean (“fixed”) pharmacokinetic parameters across a patient cohort while simultaneously quantifying inter-individual variability through “random” effects, NLMEM effectively borrow strength across subjects for optimizing the fitting. This stabilizes parameter estimates even when individual patients contribute only one or two imaging time points, thereby reducing reliance on labor-intensive multi-time-point schedules. Figure 1 illustrates the PopPK framework for simplified dosimetry.

Several clinical studies have demonstrated the feasibility and accuracy of such NLMEM-driven approaches. In peptide receptor radionuclide therapy, simplified dosimetry with NLMEM showed that a single planar scan at ∼47 h post-injection yielded a renal absorbed dose bias of 7%–8% compared to multi–time-point dosimetry, which was reduced to ∼6% when two scans at 23 and 47 h were used (25, 38). These findings provide practical guidance to physicians in selecting one- vs. two-scan protocols depending on available resources and clinical need (38). Similarly, for [^177^Lu]Lu-PSMA-617, a single SPECT/CT acquired ∼42 h post-injection produced renal absorbed dose estimates within an RMSE of ∼10% compared to full-protocol dosimetry (35), supporting the feasibility of accurate simplified dosimetry with STP imaging for routine clinical workflows.

Other studies have employed PopPK principles using Bayesian fitting approaches to enhance STP dosimetry. For example, Patrianesha et al. demonstrated that integrating population-based model selection into Bayesian fitting for [^177^Lu]Lu-PSMA-617 improved the accuracy of TIA estimation. A single SPECT/CT measurement at 48 h post-injection yielded TIA values within an RMSE of 8% compared to the reference TIA derived from multi-time-point data, highlighting the ability of Bayesian-PopPK integration to deliver reliable absorbed dose estimates under clinically constrained sampling conditions. Together, these findings underscore the clinical utility of PopPK with NLMEM for enabling accurate, reproducible, and personalized simplified dosimetry. By reducing the imaging burden, these approaches can facilitate broader implementation of individualized dosimetry in routine practice and support the transition from fixed-activity protocols toward precision-guided radiopharmaceutical therapy.

## 5 Uncertainty analysis

Uncertainty analysis is a critical component of RPT dosimetry, ensuring that patient-specific absorbed dose estimates are both reliable and clinically relevant (3, 27, 43). Jundi et al. proposed PopPK with a Bayesian fitting framework to estimate the precision of STP dosimetry in [^177^Lu]Lu-DOTATATE peptide receptor radionuclide therapy by applying a mono-exponential function to a single SPECT/CT acquisition (43). By incorporating prior distributions of the model parameters derived from multi-time-point population data fitting, their approach reliably estimated the precision of individual TIAs. STP dosimetry demonstrated lower TIA precision compared to TIA derived from multi-time-point dosimetry methods: the coefficient of variation (CV) of individual TIA standard deviations ranged from 0.8%–49% with ATP fitting, but increased to 22%–33% with STP estimates using the PopPK Bayesian fitting method (43).

Building on this, Budiansah et al. systematically assessed both the accuracy and precision of STP dosimetry using PopPK with the NLMEM framework for PRRT (16). Here, uncertainty was rigorously propagated through a PBPK model embedded within an NLMEM framework. This enabled not only accurate estimation of population and individual kinetic parameters but also inclusion of both measurement and model-related errors in the uncertainty budget. By applying the law of propagation of uncertainty, total absorbed dose uncertainty was analytically derived from the variability of individual parameter estimates and their covariance structure. This methodology is particularly advantageous for reduced imaging schedules such as STP protocols, as it transparently quantifies the additional uncertainty introduced by sparse sampling, while still providing clinically meaningful confidence bounds to guide treatment decisions.

As expected, the use of STP protocols resulted in a modestly lower precision compared to ATP fitting, as indicated by higher relative standard errors (RSEs) in both kidney and tumor absorbed dose estimates (16). Nonetheless, the availability of explicit uncertainty quantification is critical for individualized therapy planning, as it enables clinicians to gauge the robustness of absorbed dose estimates and make informed adjustments over successive treatment cycles. Such frameworks strengthen confidence in simplified dosimetry approaches, bridging the gap between clinical feasibility and scientific rigor.

## 6 Challenges and future perspectives

PopPK modeling in RPT dosimetry is hampered primarily by its computational complexity and the specialized expertise required (44). PopPK software, such as Monolix or NONMEM, requires advanced statistical knowledge and proficiency with dedicated software, skills that many nuclear medicine centers currently lack. At the same time, validating PopPK models requires comprehensive, high-quality pharmacokinetic data and robust quality-assurance protocols (44). Determining optimal sampling schedules, whether via serial imaging time points or blood draws, and establishing rigorous acceptance criteria for model performance further complicate implementation.

Another significant barrier is the scarcity of formal training and educational resources tailored to PopPK methods in the RPT context (45, 46). Most medical physicists and nuclear medicine physicians receive little to no exposure to PopPK model development or interpretation of population-based pharmacokinetic outputs. This knowledge gap not only slows the development but also undermines confidence in applying existing ones, reinforcing reliance on empirical, one-size-fits-all activity administration protocols.

Economic considerations add another layer of difficulty (45, 47). Many centers are still struggling to implement standard dosimetry workflows (48). They do not compensate for the additional time, personnel, and imaging resources required to collect the detailed data necessary for PopPK-based dosimetry. If standard dosimetry is already challenging, creating and maintaining high-quality population datasets to support PopPK modeling is even more daunting. Without financial incentives, centers lack the motivation and funding to invest in the infrastructure necessary for personalized, model-driven dosing, despite its clear long-term benefits of improved tumor control and reduced toxicity (49, 50).

Looking forward, however, several converging trends promise to dissolve these hurdles. User-friendly commercial software platforms will embed robust PopPK frameworks into intuitive graphical interfaces, allowing clinicians to perform PopPK analysis without needing to understand the underlying complexity. Simplified dosimetry based on population models will reduce the number of required scans or blood draws, ease workflow burdens, while preserving the accuracy of the estimated absorbed dose. Meanwhile, machine learning–driven automation of covariate selection, model selection, and outlier detection will enable near-real-time treatment adaptation across multi-cycle therapies. As multinational interdisciplinary collaborative consortia establish standardized PopPK libraries, quality-assured datasets, and unified regulatory guidelines, and as reimbursement policies evolve to reward personalized dosimetry, RPT dosimetry will transition from empirical practice to a precision-medicine discipline. Ultimately, clinicians will rely on streamlined software workflows that require only basic operational skills to tailor administered activities dynamically for each patient, thereby achieving truly individualized therapy.

## 7 Conclusion

PopPK modeling represents a paradigm shift in RPT dosimetry, providing a rigorous framework to individualize treatment while addressing the practical constraints of routine clinical care. PopPK modeling enables the accurate estimation of the absorbed dose from simplified imaging protocols, reducing the need for intensive multi-time-point schedules while preserving the precision required for effective treatment planning. Clinically, the impact extends beyond technical accuracy. PBMS with PopPK offers standardized and reproducible dosimetry, supporting the development of a reference framework and reducing patient and institutional burden. Furthermore, PopPK-based uncertainty analysis provides clinicians with the precision of the absorbed dose estimates, facilitating informed adjustments in multi-cycle therapy and reinforcing clinical decision-making. Looking ahead, PopPK-guided dosimetry is poised to become the foundation of precision medicine in RPT. With advances in computation and AI integration, this approach will establish individualized dosimetry as a standard of care, comparable to external beam radiotherapy treatment planning, transforming RPT from an empirical practice into a precision-driven discipline that minimizes toxicity for a prescribed absorbed dose.

## References

[1] Stokke C Gabiña PM Solný P Cicone F Sandström M Gleisner KS Dosimetry-based treatment planning for molecular radiotherapy: a summary of the 2017 report from the internal dosimetry task force. EJNMMI Phys. (2017) 4(1):27. 10.1186/s40658-017-0194-3 29164483 PMC5698234

[2] Danieli R Milano A Gallo S Veronese I Lascialfari A Indovina L Personalized dosimetry in targeted radiation therapy: a look to methods, tools and critical aspects. J Pers Med. (2022) 12(2):205. 10.3390/jpm12020205 35207693 PMC8874397

[3] O’Donoghue J Zanzonico P Humm J Kesner A. Dosimetry in radiopharmaceutical therapy. J Nucl Med. (2022) 63(10):1467–74. 10.2967/jnumed.121.262305 36192334 PMC12079709

[4] Wehrmann C Senftleben S Zachert C Müller D Baum RP. Results of individual patient dosimetry in peptide receptor radionuclide therapy with ^177^Lu DOTA-TATE and ^177^Lu DOTA-NOC. Cancer Biother Radiopharm. (2007) 22(3):406–16. 10.1089/cbr.2006.325 17651048

[5] Flux GD Chittenden SJ Saran F Gaze MN. Clinical applications of dosimetry for mIBG therapy. Q J Nucl Med Mol Imaging. (2011) 55(2):116–25. PMID: 21386786 21386786

[6] Strigari L Konijnenberg M Chiesa C Bardies M Du Y Gleisner KS The evidence base for the use of internal dosimetry in the clinical practice of molecular radiotherapy. Eur J Nucl Med Mol Imaging. (2014) 41(10):1976–88. 10.1007/s00259-014-2824-5 24915892

[7] Hardiansyah D Begum NJ Kletting P Mottaghy FM Glatting G. Sensitivity analysis of a physiologically based pharmacokinetic model used for treatment planning in peptide receptor radionuclide therapy. Cancer Biother Radiopharm. (2016) 31(6):217–24. 10.1089/cbr.2016.2012 27403777

[8] Maccauro M Cuomo M Bauckneht M Bagnalasta M Mazzaglia S Scalorbi F The LUTADOSE trial: tumour dosimetry after the first administration predicts progression free survival in gastro-entero-pancreatic neuroendocrine tumours (GEP NETs) patients treated with [^177^Lu]Lu-DOTATATE. Eur J Nucl Med Mol Imaging. (2024) 52(1):291–304. 10.1007/s00259-024-06863-y 39235614

[9] Hebert K Santoro L Monnier M Castan F Berkane I Assénat E Absorbed dose-response relationship in patients with gastroenteropancreatic neuroendocrine tumors treated with [^177^Lu]lu-DOTATATE: one step closer to personalized medicine. J Nucl Med. (2024) 65(6):923–30. 10.2967/jnumed.123.267023 38637144 PMC11149595

[10] Warfvinge CF Gustafsson J Roth D Tennvall J Svensson J Bernhardt P Relationship between absorbed dose and response in neuroendocrine tumors treated with [^177^Lu]Lu-DOTATATE. J Nucl Med. (2024) 65(7):1070–5. 10.2967/jnumed.123.266991 38724277

[11] Deshayes E Karfis I Santoro L Mileva M Danieli R Hebert K Patient-specific dosimetry-driven PRRT: time to move forward!. J Nucl Med. (2025) 66(6):983. 10.2967/jnumed.124.268880 40210422

[12] Grkovski M Krebs SS O'Donoghue JA Kuten J Mauguen A Shobeiri P Lesion absorbed dose-response relationship in patients with metastatic castration-resistant prostate cancer undergoing [^177^Lu]Lu-PSMA-617 radiopharmaceutical therapy. J Nucl Med. (2025) 66:1622–30. 10.2967/jnumed.125.270170 40774698 PMC12487730

[13] Council of the European U. Council directive 2013/59/euratom of 5 december 2013 laying down basic safety standards for protection against the dangers arising from exposure to ionising radiation, and repealing directives 89/618/euratom, 90/641/euratom, 96/29/euratom, 97/43/euratom and 2003/122/euratom. Off J Eur Union. (2014) 13:1–73. https://eur-lex.europa.eu/eli/dir/2013/59/oj/eng

[14] Patrianesha BB Peters SMB Hardiansyah D Ritawidya R Prive BM Nagarajah J Single-time-point dosimetry using model selection and the Bayesian fitting method: a proof of concept. Phys Med. (2025) 129:104868. 10.1016/j.ejmp.2024.104868 39642576

[15] Hardiansyah D Riana A Hänscheid H Beer AJ Lassmann M Glatting G. Non-linear mixed-effects modelling and population-based model selection for ^131^I kinetics in benign thyroid disease. EJNMMI Phys. (2025) 12(1):37. 10.1186/s40658-025-00735-6 40198532 PMC11979076

[16] Budiansah I Hardiansyah D Riana A Pawiro SA Beer AJ Glatting G. Accuracy and precision analyses of single-time-point dosimetry utilising physiologically-based pharmacokinetic modelling and non-linear mixed-effects modelling. EJNMMI Phys. (2025) 12(1):26. 10.1186/s40658-025-00726-7 40138034 PMC11947405

[17] Devasia TP Dewaraja YK Frey KA Wong KK Schipper MJ. A novel time–activity information-sharing approach using nonlinear mixed models for patient-specific dosimetry with reduced imaging time points: application in SPECT/CT after ^177^Lu-DOTATATE. J Nucl Med. (2021) 62(8):1118–25. 10.2967/jnumed.120.256255 33443063 PMC8833869

[18] FDA. Guidance for Industry: Population Pharmacokinetics (1999).

[19] EMA. Guideline on Reporting the Results of Population Pharmacokinetic Analyses. London, UK: CHMP (2007).

[20] Hardiansyah D Maass C Attarwala AA Muller B Kletting P Mottaghy FM The role of patient-based treatment planning in peptide receptor radionuclide therapy. Eur J Nucl Med Mol Imaging. (2016) 43(5):871–80. 10.1007/s00259-015-3248-6 26577941

[21] Merrill S Horowitz J Traino AC Chipkin SR Hollot CV Chait Y. Accuracy and optimal timing of activity measurements in estimating the absorbed dose of radioiodine in the treatment of Graves’ disease. Phys Med Biol. (2011) 56(3):557–71. 10.1088/0031-9155/56/3/003 21212469

[22] Puszkiel A Bauriaud-Mallet M Bourgeois R Dierickx L Courbon F Chatelut E. Evaluation of the interaction of amino acid infusion on ^177^Lu-dotatate pharmacokinetics in patients with gastroenteropancreatic neuroendocrine tumors. Clin Pharmacokinet. (2019) 58(2):213–22. 10.1007/s40262-018-0674-1 29736841

[23] Hänscheid H Lapa C Buck AK Lassmann M Werner RA. Dose mapping after endoradiotherapy with (177)Lu-DOTATATE/DOTATOC by a single measurement after 4 days. J Nucl Med. (2018) 59(1):75–81. 10.2967/jnumed.117.193706 28588150

[24] Madsen MT Menda Y O'Dorisio TM O'Dorisio MS. Technical note: single time point dose estimate for exponential clearance. Med Phys. (2018) 45(5):2318–24. 10.1002/mp.12886 29577338 PMC5948162

[25] Hardiansyah D Riana A Beer AJ Glatting G. Single-time-point dosimetry using model selection and nonlinear mixed-effects modelling: a proof of concept. EJNMMI Phys. (2023) 10(1):12. 10.1186/s40658-023-00530-1 36759362 PMC9911583

[26] Sjögreen Gleisner K Chouin N Gabina PM Cicone F Gnesin S Stokke C EANM dosimetry committee recommendations for dosimetry of ^177^Lu-labelled somatostatin-receptor- and PSMA-targeting ligands. Eur J Nucl Med Mol Imaging. (2022) 49(6):1778–809. 10.1007/s00259-022-05727-7 35284969 PMC9015994

[27] Gear JI Cox MG Gustafsson J Gleisner KS Murray I Glatting G EANM practical guidance on uncertainty analysis for molecular radiotherapy absorbed dose calculations. Eur J Nucl Med Mol Imaging. (2018) 45(13):2456–74. 10.1007/s00259-018-4136-7 30218316 PMC6208822

[28] Hardiansyah D Riana A Kletting P Zaid NRR Eiber M Pawiro SA A population-based method to determine the time-integrated activity in molecular radiotherapy. EJNMMI Phys. (2021) 8(1):82. 10.1186/s40658-021-00427-x 34905131 PMC8671591

[29] Steiner J Nguyen B Jafari F. A pharmacokinetic model determination of time activity curves in radiopharmaceutical therapy. Mol Imaging. (2024) 23:15353508241280015. 10.1177/15353508241280015 40098749 PMC11911383

[30] Brosch-Lenz J Delker A Volter F Unterrainer LM Kaiser L Bartenstein P Toward single-time-point image-based dosimetry of ^177^Lu-PSMA-617 therapy. J Nucl Med. (2023) 64(5):767–74. 10.2967/jnumed.122.264594 36657980 PMC10152120

[31] Kurkowska S Brosch-Lenz J Dewaraja YK Frey E Sunderland J Uribe C. An international study of factors affecting variability of dosimetry calculations, part 4: impact of fitting functions in estimated absorbed doses. J Nucl Med. (2025) 66(3):441–8. 10.2967/jnumed.124.268612 39978818 PMC11876727

[32] Grob D Privé BM Muselaers CHJ Mehra N Nagarajah J Konijnenberg MW Bone marrow dosimetry in low volume mHSPC patients receiving Lu-177-PSMA therapy using SPECT/CT. EJNMMI Phys. (2024) 11(1):34. 10.1186/s40658-024-00636-0 38568428 PMC10991600

[33] Kletting P Schimmel S Kestler HA Hanscheid H Luster M Fernandez M Molecular radiotherapy: the NUKFIT software for calculating the time-integrated activity coefficient. Med Phys. (2013) 40(10):102504. 10.1118/1.4820367 24089925

[34] Ivashchenko OV O'Doherty J Hardiansyah D Cremonesi M Tran-Gia J Hippelainen E Time-activity data fitting in molecular radiotherapy: methodology and pitfalls. Phys Med. (2024) 117:103192. 10.1016/j.ejmp.2023.103192 38052710

[35] Hardiansyah D Yousefzadeh-Nowshahr E Kind F Beer AJ Ruf J Glatting G Single-time-point renal dosimetry using nonlinear mixed-effects modeling and population-based model selection in [^177^Lu]Lu-PSMA-617 therapy. J Nucl Med. (2024) 65(4):566–72. 10.2967/jnumed.123.266268 38423787

[36] Hardiansyah D Riana A Eiber M Beer AJ Glatting G. Population-based model selection for an accurate estimation of time-integrated activity using non-linear mixed-effects modelling. Z Med Phys. (2024) 34(3):419–27. 10.1016/j.zemedi.2023.01.007 36813594 PMC11384081

[37] Hardiansyah D Riana A Beer AJ Glatting G. Single-time-point estimation of absorbed doses in PRRT using a non-linear mixed-effects model. Z Med Phys. (2023) 33(1):70–81. 10.1016/j.zemedi.2022.06.004 35961809 PMC10082376

[38] Subangun RM Hardiansyah D Ibrahim RFI Patrianesha BB Hidayati NR Beer AJ Few-time-points time-integrated activity coefficients calculation using non-linear mixed-effects modeling: proof of concept for [^111^In]In-DOTA-TATE in kidneys. Phys Med. (2025) 129:104865. 10.1016/j.ejmp.2024.104865 39631133

[39] Mould DR Upton RN. Basic concepts in population modeling, simulation, and model-based drug development-part 2: introduction to pharmacokinetic modeling methods. CPT Pharmacometrics Syst Pharmacol. (2013) 2(4):e38. 10.1038/psp.2013.14 23887688 PMC3636497

[40] Upton RN Mould DR. Basic concepts in population modeling, simulation, and model-based drug development: part 3-introduction to pharmacodynamic modeling methods. CPT Pharmacometrics Syst Pharmacol. (2014) 3(1):e88. 10.1038/psp.2013.71 24384783 PMC3917320

[41] Upton RN Foster DJ Abuhelwa AY. An introduction to physiologically-based pharmacokinetic models. Pediatric Anesthesia. (2016) 26(11):1036–46. 10.1111/pan.12995 27550716

[42] Siebinga H de Wit-van der Veen BJ de Vries-Huizing DMV Vogel WV Hendrikx J Huitema ADR. Quantification of biochemical PSA dynamics after radioligand therapy with [^177^Lu]Lu-PSMA-I&T using a population pharmacokinetic/pharmacodynamic model. EJNMMI Phys. (2024) 11(1):39. 10.1186/s40658-024-00642-2 38656678 PMC11043318

[43] Jundi AF Naqiyyun MD Patrianesha BB Mu'minah IAS Riana A Hardiansyah D. Uncertainty analysis of time-integrated activity coefficient in single-time-point dosimetry using Bayesian fitting method. Nucl Med Mol Imaging. (2024) 58(3):120–8. 10.1007/s13139-024-00851-8 38633290 PMC11018592

[44] Siebinga H de Wit-van der Veen BJ Stokkel MDM Huitema ADR Hendrikx J. Current use and future potential of (physiologically based) pharmacokinetic modelling of radiopharmaceuticals: a review. Theranostics. (2022) 12(18):7804–20. 10.7150/thno.77279 36451855 PMC9706588

[45] Divgi C Carrasquillo JA Meredith R Seo Y Frey EC Bolch WE Overcoming barriers to radiopharmaceutical therapy (RPT): an overview from the NRG-NCI working group on dosimetry of radiopharmaceutical therapy. Int J Radiat Oncol Biol Phys. (2021) 109(4):905–12. 10.1016/j.ijrobp.2020.12.002 33309909 PMC8399328

[46] Scott AM Zeglis BM Lapi SE Scott PJH Windhorst AD Abdel-Wahab M Trends in nuclear medicine and the radiopharmaceutical sciences in oncology: workforce challenges and training in the age of theranostics. Lancet Oncol. (2024) 25(6):e250–e9. 10.1016/S1470-2045(24)00037-8 38821099 PMC11345887

[47] Adedapo KS Onimode YA Ejeh JE Adepoju AO. Avoidable challenges of a nuclear medicine facility in a developing nation. Indian J Nucl Med. (2013) 28(4):195–9. 10.4103/0972-3919.121962 24379527 PMC3866662

[48] Peters S Tran-Gia J Agius S Ivashchenko OV Badel JN Cremonesi M Implementation of dosimetry for molecular radiotherapy; results from a European survey. Phys Med. (2024) 117:103196. 10.1016/j.ejmp.2023.103196 38104033

[49] Flux G Buscombe J. BNMS position statement on molecular radiotherapy. Nucl Med Commun. (2021) 42(10):1061–3. 10.1097/MNM.0000000000001458 34284439 PMC8445360

[50] Fisher DR. Perspectives on internal dosimetry for optimized radionuclide therapy. Cancer Biother Radiopharm. (2022) 37(3):161–3. 10.1089/cbr.2021.0318 34569812

