# Supplementary material for: Population pharmacokinetic modeling in radiopharmaceutical therapy: a review
Source: Front Nucl Med. 2025 Oct 9;5:1695332. doi: 10.3389/fnume.2025.1695332 (PMC12546074; doi:10.3389/fnume.2025.1695332)
Supplement: Supplementary file 1 [file Datasheet1.pdf]

Supplementary Material

Population Pharmacokinetic Modeling in Radiopharmaceutical Therapy: A Review

Deni Hardiansyah[1], Bisma Barron Patrianesha[2], Gerhard Glatting[3]

[1]*Faculty of Mathematics and Natural Sciences, Medical Physics and Biophysics Division, Physics Department, Universitas Indonesia, Depok, Indonesia*

[2]*Research Center for Safety, Metrology, and Nuclear Quality Technology, National Research and Innovation Agency (BRIN), KST B.J. Habibie, Tangerang Selatan, Indonesia*

[3]*Medical Radiation Physics, Department of Nuclear Medicine, Ulm University, Ulm, Germany*

Examples of PopPK studies

PopPK modeling has been increasingly applied in radiopharmaceutical therapy to optimize treatment strategies, improve response prediction, and enhance patient safety. The following table summarizes representative studies categorized into pharmacodynamic analysis, simplified dosimetry approaches, and uncertainty analysis. Each study highlights the methodological approaches used, key findings, and potential clinical implications, illustrating the growing role of PopPK and modeling techniques in advancing personalized radiopharmaceutical therapy.

Table S1. Examples of PopPK studies

No	Title and Authors	Methodological Approaches	Key Findings	Clinical implications
**Pharmacodynamic Analysis**				
1	Evaluation of the Interaction of Amino Acid Infusion on ^{177}Lu-Dotatate Pharmacokinetics in Patients with Gastroenteropancreatic Neuroendocrine Tumors.<br><br>(Puszkiel et al., 2019, *Clin Pharmacokinet*)	Two- and three-compartment models were developed to describe the pharmacokinetics of [^{177}Lu]Lu-DOTATATE in the blood samples of 42 patients after amino acid infusion. Covariate analysis was performed to assess the influence of covariates on the pharmacokinetic parameters, with model performance checked by fit plots, bootstrap, and pcVPC. The relationship between drug exposure and renal and hematologic toxicity was assessed using linear regression, while nonparametric tests were used to determine significant changes in biological variables over time.	Amino acid co-infusion had a significant impact on ^{177}Lu-DOTATATE pharmacokinetics.<br><br>The inter-individual variability of the amino acid effect was high, causing different responses among patients.	The high inter-individual variability in amino acid effects suggests that current one-size-fits-all approaches to nephroprotection may not be optimal for all patients. Individualized dosing strategies based on patient-specific pharmacokinetic responses are needed.
2	Quantification of biochemical PSA dynamics after radioligand therapy with	A population PK model was built using quantitative SPECT/CT data from 406 scans of 76 patients treated with multiple	Tumor volume, kidney function, and the number of treatment cycles significantly influence	By using PK/PD analysis, clinicians can select patients to determine who is likely to

	[177Lu]Lu-PSMA-I&T using a population pharmacokinetic/ pharmacodynamic model<br><br>(Siebinga et al., 2023, *EJNMMI Phys*)	cycles of [177Lu]Lu-PSMA-I&T (±7.4 GBq, every 2 or 6 weeks). The model included five compartments: central, salivary glands, kidneys, tumors, and other tissues. Covariates such as tumor volume, renal function, and cycle number were tested to explain variability in uptake. The model was then expanded with a PD compartment to describe PSA changes during and after treatment. To assess exposure–response, estimated tumor concentrations of [177Lu]Lu-PSMA-I&T were related to PSA dynamics over time.	the amount of drug that reaches the tumors and, consequently, how well a patient responds.	benefit from the therapy and monitor their response over time.
**Simplified Dosimetry**				
3	Single-time-point dosimetry using model selection and nonlinear mixed-effects modelling: a proof of concept<br><br>(Hardiansah et al., 2018, *EJNMMI Phys*)	The methodology involves acquiring all-time-point (ATP) biokinetic data using planar imaging at several time points post-injection for a radiopharmaceutical, such as [111In]In-DOTATATE, from 8 patients. The [111In]In-DOTATATE biokinetic was used as the surrogate of peptide receptor radionuclide therapy using [90Y]Y-DOTATATE. These data are used to establish a population-based nonlinear mixed-effects (NLME) model, which simultaneously fits fixed and random effects, with model selection performed using Akaike weights to identify the best fit function. The accuracy of the single-time-point (STP) dosimetry approach is	Population-based model selection using non-linear mixed effects helps identify the best function. It provides better accuracy for calculating time-integrated activity coefficient (TIACs) than commonly used functions, such as the mono-exponential function.	STP dosimetry offers benefits, including reduced patient burden and cost-effectiveness. Personalized dosimetry in radiopharmaceutical therapy (RPT) can be more practical and accessible using STP dosimetry.

		then evaluated by comparing time-integrated activities (TIA) calculated using the optimal STP model against TIAs from the ATP dosimetry.		
4	Few-time-points time-integrated activity coefficients calculation using non-linear mixed-effects modeling: Proof of concept for [^{111}In]In-DOTA-TATE in kidneys<br><br>(Subangun et al. 2025, *Phys Med*)	The [^{111}In]In-DOTATATE biokinetic was used as the surrogate of peptide receptor radionuclide therapy using [^{90}Y]Y-DOTATATE. The methodology involved establishing a reference TIAC (rTIAC) by fitting a four-parameter sum-of-exponentials function to data from all five measurement time points. This was followed by calculating estimated TIACs (eTIACs) for all combinations of fewer time points (one, two, three, and four) using the same NLME model. To assess the accuracy of the few-time-point method, the eTIACs were then compared to the rTIACs by calculating relative deviations and root-mean-square errors, revealing that accuracy varied based on the specific time points used.	The accuracy depends on the combination of time points that are used. The most accurate estimations were achieved when biokinetic data from a later time point were included.	Dosimetry with a few time points offers more flexible treatment management, as an estimate can still be calculated even if a planned time point is missed.
5	Single-Time-Point Renal Dosimetry Using Nonlinear Mixed-Effects Modeling and Population-Based Model Selection in [^{177}Lu]Lu-PSMA-617 Therapy	Biokinetic data of [^{177}Lu]Lu-PSMA-617 in the kidneys were collected from 63 patients at five time points using SPECT/CT. Thirteen exponential models were tested, and the best was selected with NLME modeling. This model provided reference absorbed doses. STP dosimetry was then estimated by combining patient	The NLME-based STP method outperformed other existing STP methods, such as those by Hänscheid and Madsen.	This approach makes individualized dosimetry more accessible and likely to be adopted in routine clinical practice, ultimately benefiting patients by enabling better

	(Hardiansyah et al., 2024, *J Nucl Med*)	STP data with population ATP data. Accuracy was assessed with relative deviation (RD) and root mean squared error (RMSE), and the method was compared with the Hänscheid and Madsen approaches.		risk prediction for treatment side effects.
6	Single-time-point dosimetry using model selection and the Bayesian fitting method: A proof of concept<br><br>(Patrianesha et al., 2025, *Phys Med*)	Population-based model selection was used to identify the most suitable function for the kidneys of 10 patients who received [^{177}Lu]Lu-PSMA-617 therapy. The Bayesian fitting method was used to estimate the TIACs with only STP imaging. Accuracy was assessed with RD and RMSE.	The Bayesian fitting method for STP dosimetry outperformed other existing STP Hänscheid methods. The use of a mono-exponential function is not always suitable for calculating TIACs.	
**Uncertainty analysis**				
7	Uncertainty Analysis of Time-Integrated Activity Coefficient in Single-Time-Point Dosimetry Using Bayesian Fitting Method (Jundi et al., 2018, *Nucl Med Mol Imaging*)	The Bayesian fitting (BF) approach was developed to quantify uncertainty in STP dosimetry. The method utilized an extended objective function incorporating prior knowledge of fitting parameters. Reference TIACs were first determined from all-time-point data. Two BF methods, relative-based (BFr) and absolute-based (BFa), were then applied to estimate TIACs from STP data. Performance was evaluated by comparing calculated TIACs to the reference values,	The Bayesian Fitting method can be used to calculate the uncertainty of individual TIAC in STP dosimetry.<br><br>The uncertainty of the individual STP-calculated absorbed doses was lower than that of the ATP method.	Increased reliability of the calculated absorbed dose in the kidneys for optimizing treatment planning.

		and uncertainty was calculated using the jackknife method with error propagation.		
8	Accuracy and precision analyses of single-time-point dosimetry utilising physiologically-based pharmacokinetic modelling and non-linear mixed-effects modelling.<br><br>(Budiansah et al. 2025, *EJNMMI Phys*)	Utilizing physiologically-based pharmacokinetic (PBPK) and non-linear mixed-effects (NLME) modeling, this analysis assessed STP dosimetry. The PBPK/NLME model, developed from multi-time-point patient data, was used to estimate absorbed doses using only one measurement per patient. Accuracy was evaluated by comparing STP estimates with full multi-time-point results, while precision was determined by measuring estimate uncertainty.	The uncertainty of the STP-calculated absorbed doses was consistently lower than that of the ATP method across all patients.	Increased accuracy and reliability of the calculated absorbed dose in various organs for optimizing treatment planning.

Example Software Platforms for Population Pharmacokinetic (PopPK) Analysis

A wide range of software platforms is available to support population pharmacokinetic (PopPK) analysis, each offering distinct features, licensing models, and target user groups. Commercial software, such as NONMEM, Monolix, Phoenix WinNonlin, Simcyp, and MATLAB SimBiology, is widely recognized in the pharmaceutical industry for its robustness, regulatory acceptance, and technical support. In parallel, academic freeware such as ADAPT 5 and PKSolver provides accessible options for research and teaching without licensing costs, though often with more limited functionality. Additionally, several open-source tools, including nlmixr, saemix, mrgsolve, PKPDsim, Stan, and Torsten, offer flexible and transparent modeling environments that are particularly attractive for academic research and methodological development.

The following table summarizes representative software solutions for PopPK analysis, categorized into **commercial**, **academic freeware**, and **open-source** platforms, along with their main strengths, typical applications, and access information.

Table S.2. The Commercial and Open-source Software for PopPK Analysis

Category	Software	Strengths	Typical Use Context	Company / Origin / Website
Commercial	NONMEM	Robust, industry-standard, regulator-accepted, highly flexible modeling	Regulated drug development, pharmaceutical industry	ICON plc, Dublin, Ireland → www.iconplc.com
	Monolix	User-friendly interface, strong technical support, supports modern methods	Pharmaceutical industry, applied research	Lixoft (part of Certara), Antony, France → lixoft.com
	Phoenix WinNonlin	User-friendly GUI, many add-on modules, comprehensive documentation	Pharmacokinetic analysis in industry	Certara, Princeton, New Jersey, USA → www.certara.com
	Simcyp Simulator	Advanced PBPK population modeling, drug–drug interaction prediction, variability analysis	Drug development, regulatory submissions	Certara, Sheffield, UK → www.certara.com/simcyp-simulator

	MATLAB SimBiology and nlme toolbox	Graphical and script-based modeling for PK/PD, PBPK, and systems biology; strong simulation capabilities. Widely available in academia via institutional licenses.	Academic research, teaching, applied PK/PD modeling	MathWorks, Natick, Massachusetts, USA → https://www.mathworks.com/help/simbio/nonlinear-mixed-effects-estimation-simbiology.html
Academic / Proprietary Freeware	ADAPT 5	Flexible PK/PD modeling and simulation platform, supports population approaches	Academic and industry PK/PD research	Biomedical Simulations Resource, University of Southern California, USA → bmsr.usc.edu/software/adapt
	PKSolver	Excel add-in, simple interface for basic PK/PD modeling	Academic teaching, small-scale analyses	Nanjing University of Chinese Medicine, China → https://doi.org/10.1016/j.cmpb.2010.01.007
Open-source	nlmixr<br><br>(R package)	Free, flexible, integrated with the R ecosystem	Academic research, methodological exploration	Open-source community → CRAN & GitHub
	saemix<br><br>(R package)	Free, SAEM implementation for PopPK, lightweight, and easy integration	Academia, basic research	Open-source community → CRAN & GitHub
	mrgsolve<br><br>(R package)	ODE-based population modeling, NONMEM-like syntax, integrates with R	Academic/industry hybrid modeling	Open-source community → mrgsolve.github.io
	PKPDsim<br><br>(R package)	Flexible PK/PD simulations, works with nlmixr	Academic simulation studies	Open-source community → CRAN

	Stan	General Bayesian inference platform, very flexible, supports hierarchical models	Bayesian PK/PD, PopPK, complex modeling	Stan Development Team, USA/Global → Stan
	Torsten	Stan extension with specialized PK/PD functions	Bayesian PK/PD population analysis	Open-source community (Metrum Research Group & collaborators) → metrumresearchgroup.github.io/Torsten
